# Preoperative Butyrylcholinesterase Level as an Independent Predictor of Overall Survival in Clear Cell Renal Cell Carcinoma Patients Treated with Nephrectomy

**DOI:** 10.1155/2014/948305

**Published:** 2014-03-04

**Authors:** Takuya Koie, Chikara Ohyama, Jotaro Mikami, Hiromichi Iwamura, Naoki Fujita, Tendo Sato, Yuta Kojima, Ken Fukushi, Hayato Yamamoto, Atsushi Imai, Shingo Hatakeyama, Takahiro Yoneyama, Yasuhiro Hashimoto, Masato Kitayama, Kazuyoshi Hirota

**Affiliations:** ^1^Department of Urology, Hirosaki University Graduate School of Medicine, Zaifucho 5, Hirosaki 036-8562, Japan; ^2^Department of Anesthesiology, Hirosaki University Graduate School of Medicine, Zaifucho 5, Hirosaki 036-8562, Japan

## Abstract

The prognostic factors for the overall survival (OS) of clear cell renal cell carcinoma (ccRCC) patients treated with nephrectomy are not well defined. In the present study, we investigated the prognostic significance of preoperative butyrylcholinesterase (BChE) levels in 400 ccRCC patients undergoing radical or partial nephrectomy from 1992 to 2013 at our institution. Univariate and multivariate analyses were performed to determine the clinical factors associated with OS. Among the enrolled patients, 302 were diagnosed with organ-confined disease only (T1-2N0M0), 16 with lymph node metastases, and 56 with distant metastases. The median preoperative BChE level was 250 U/L (normal range, 168–470 U/L), and median follow-up period was 36 months. The 3-year OS rate in patients with preoperative BChE levels of ≥100 U/L was significantly higher than in those with levels of <100 U/L (89.3% versus 77.7%, *P* = 0.004). On univariate analysis, performance status; anemia; hypoalbuminemia; preoperative levels of BChE, corrected calcium, and C-reactive protein; and distant metastasis status were significantly associated with OS. Multivariate analysis revealed that preoperative BChE levels and distant metastasis status were significantly associated with OS. Our findings suggest a possible role of preoperative BChE levels as an independent predictor of OS after nephrectomy in ccRCC patients.

## 1. Introduction

Despite an increase in early diagnosis owing to improved imaging technologies, up to one-third of renal cell carcinoma (RCC) patients are diagnosed with the disease at metastatic stages [1]. Of the remaining two-thirds, approximately 20%–40% of those treated with nephrectomy develop metachronous metastases or local recurrence [2]. Therefore, it is essential to accurately stratify patients according to their overall survival (OS) or recurrence-free survival (RFS) after surgery. Several clinical, pathological, and molecular markers such as C-reactive protein (CRP), preoperative platelet count, and plasma fibrinogen level have enabled more accurate prognosis in RCC [3–6]. However, in general, prognostic factors for OS of RCC patients, particularly those with clear cell RCC (ccRCC), treated with nephrectomy remain poorly defined.

Systemic inflammation is a common host reaction to carcinogenesis or cancer progression [7]. Butyrylcholinesterase (BChE) is an alpha-glycoprotein present in many tissue types, including the central and peripheral nervous system, and in the liver. Low BChE serum levels have been reported in many clinical conditions such as liver damage, inflammation, injury, and malnutrition [8]. A decrease in pretreatment serum BChE level has also been suggested as a useful prognostic parameter in advanced cancer patients with or without hepatic involvement [9–12]. However, the predictive value of serum BChE level in kidney cancer patients is unknown. Thus, in the present study, we aimed to evaluate the prognostic significance of pretreatment BChE levels in patients with ccRCC undergoing nephrectomy.

## 2. Materials and Methods

### 2.1. Patients

In the present study, we reviewed the clinical charts of 551 patients who underwent partial, radical, or cytoreductive nephrectomy between 1992 and 2013 at a single institution. Our analysis focused on 400 patients who were diagnosed with ccRCC postoperatively. Covariates including age; gender; Eastern Cooperative Oncology Group performance status (PS); preoperative laboratory data on serum levels of hemoglobin, albumin, BChE, corrected calcium (Ca), lactate dehydrogenase (LDH), CRP, and neutrophil-lymphocyte ratio (NLR); clinical T stage; and distant metastasis status were analyzed. All laboratory data were routinely collected before surgery in all patients. Tumor staging was performed according to the American Joint Committee on Cancer Staging Manual [13]. The study protocol and informed consent documents were reviewed and approved by the Hirosaki University institutional review board.

### 2.2. Follow-Up Schedule

After nephrectomy, patients with T1 disease received annual followup, whereas those with T2/T3/T4 status were evaluated every 3 months for 2 years and 6 months thereafter. During the follow-up visits, patients underwent physical examination, ultrasonography, and computed tomography (CT).

### 2.3. Endpoints and Statistical Analysis

The endpoint in this study was overall survival. Data were analyzed using SPSS 22 statistical software (IBM Corp., Armonk, NY, USA). Continuous variables were presented with the median value and interquartile range. Survival after nephrectomy was analyzed using the Kaplan-Meier method. Comparison of subgroup survival was performed using the log-rank test. The covariates examined in the univariate analysis were PS (0 versus ≥1), hemoglobin level (≥lower limit of normal (LLN) versus <LLN), albumin level (≥LLN versus <LLN), BChE level (≥100 U/L versus <100 U/L), NLR (≤2.5 versus >2.5), corrected Ca level (≤10 mg/dL versus >10 mg/dL), LDH (≤1.5 × upper limit of normal (ULN) versus >1.5 × ULN), CRP (≤0.3 mg/dL versus >0.3 mg/dL), T stage (T1 versus T2 versus T3 versus T4), lymph node status (N0 versus N1), and metastasis status (M0 versus M1). Multivariate analysis was performed using the Cox proportional hazard model. All *P* values were 2-sided, and the significance level was set at <0.05.

## 3. Results

### 3.1. Patient Characteristics

The pretreatment characteristics of all patients are listed in Table 1. All diagnoses were conducted based on CT findings. Among the enrolled patients, 302 were diagnosed with organ-confined disease (T1-2N0 M0), whereas 56 had a distant metastasis (any T, any N, and M1). The most frequent metastasis sites were the lungs (36 patients, 64%) and the bone (9 patients, 16%).

### 3.2. Oncological Outcomes

**Figure 1:**
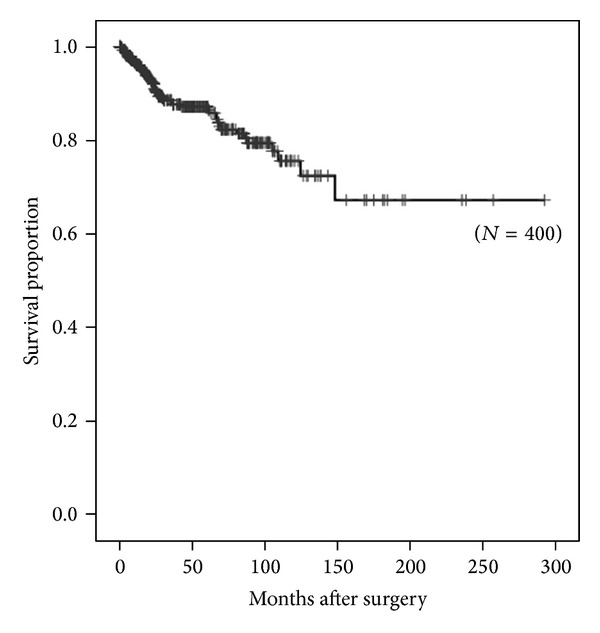
Kaplan-Meier estimate of overall survival. The 3-year overall survival rate was 88.6%.

The OS was 88.6% for all 400 enrolled patients (Figure 1). By the end of the follow-up period, 38 patients (10%) had died of RCC, and 17 (4%) had died of other causes. Nineteen patients (5%) remained alive despite metastases from ccRCC.

**Figure 2:**
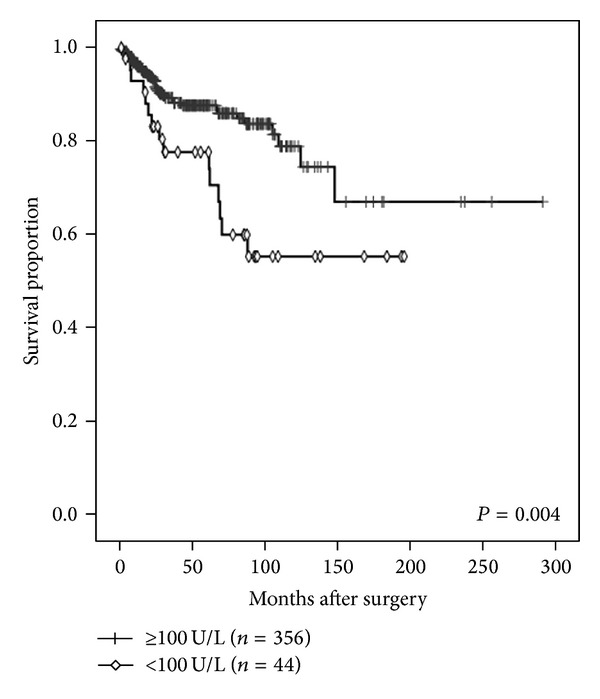
Kaplan-Meier estimate of overall survival (OS) according to serum butyrylcholinesterase (BChE) levels. The 3-year OS rate was 89.3% in patients with preoperative BChE levels of ≥100 U/L and 77.7% in those with preoperative BChE levels of <100 U/L (*P* = 0.004).

On univariate analysis, the statistically significant prognostic factors were PS (*P* = 0.003), hemoglobin level (*P* = 0.004), albumin level (*P* = 0.010), BChE level (*P* < 0.001), corrected Ca level (*P* = 0.007), CRP level (*P* < 0.001), clinical T stage (*P* < 0.001), and metastasis status (*P* < 0.001), as shown in Table 2. Kaplan-Meier curves for OS of all patients, stratified by different serum BChE levels, are shown in Figure 2.

Multivariate analysis using the Cox proportional hazard model revealed that metastasis status and serum BChE level were independent prognostic predictors for OS (Table 3). No significant relation was noted between the BChE level and distant metastasis status.

## 4. Discussion

Results from the present study validate the possible role of preoperative serum BChE levels as an independent prognostic factor for OS after surgery in ccRCC patients. To our knowledge, this retrospective analysis is the first report to evaluate the prognostic significance of pretreatment serum BChE levels in ccRCC patients.

Cholinesterases are a group of enzymes that hydrolyze acetylcholine and other choline esters. There are 2 main types of cholinesterases with different biochemical properties [14]. Acetylcholinesterase is present in all excitable tissues—such as the central and peripheral nerve systems and muscles—and erythrocytes. The other cholinesterase, BChE, is also present in the nervous system as well as the liver. As BChE is synthesized in the liver, a hepatocellular impairment would result in a decreased activity of the enzyme. In fact, reduction of BChE levels often occurs in acute and chronic liver damage, cirrhosis, and liver metastasis. Similarly, low BChE levels have been reported during stress and inflammation, as well as in cases of protein-energy malnutrition and other clinical conditions [8].

In geriatric patients, BChE levels have been suggested as a useful biomarker for malnutrition or a disease prognostic indicator [15]. Levels of CRP, interleukin 6 (IL-6), and tumor necrosis factor alpha (TNF-alpha) have been reported to significantly increase in frail elderly patients, whereas BChE activity has been observed to significantly decrease (*P* < 0.005) [14]. In addition, BChE activity has been reported to negatively correlate with IL-6 and TNF-alpha levels [14].

Advanced cancer is a clinical condition involving mild to moderate inflammation. Systemic inflammation has been described to be associated with poor prognosis in a variety of malignancies [16]. Plasma BChE levels have been shown to decrease in advanced cancer patients with or without hepatic involvement, despite the other liver function tests yielding normal results [17]. One of the possible mechanisms for such BChE activity decrease in cancer patients could be secondary anorexia accompanying malignancy [18]. Santarpia et al. suggested that besides albumin levels and Karnofsky index, serum BChE levels were a survival predictive factor in terminal cancer patients with peritoneal carcinomatosis [9]. In another study involving patients with head and neck or uterine cervical cancer, BChE activity was demonstrated to be an effective prognostic marker [10].

The present study has limitations inherent to any retrospective analysis with a limited number of patients. Although 76% of the enrolled patients were diagnosed with organ-confined disease, the other 24% had an advanced disease with metastases to the lymph nodes or other distant sites. BChE is a sensitive yet nonspecific serum biomarker. Therefore, a decreased BChE level might result from an inflammation or physical stress. In addition, the correlation between the decrease of serum BChE level and advanced disease or distant metastasis was obscure in this study. However, in ccRCC patients, low serum BChE levels could suggest other systemic disorders, including poor PS or secondary malnutrition. In addition, BChE levels seem to correlate with cancer activity and nutritional status in ccRCC patients.

## 5. Conclusion

Our results validate the possible role of preoperative serum BChE levels as an independent prognostic factor after surgery in ccRCC. BChE levels may correlate with cancer activity and nutritional status in ccRCC patients. Therefore, our findings suggest that serum BChE assessment be included in the routine clinical evaluation of patients with ccRCC.

## Figures and Tables

**Table 1:** Patient characteristics.

Age (years, median, IQR)	65 (56–73)
Gender (*N*, %)	
Male	286 (72)
Female	114 (28)
Performance status (*N*, %)	
0	368 (92)
≥1	32 (8)
Clinical T (*N*, %)	
T1	261 (65)
T2	41 (10)
T3	88 (22)
T4	10 (3)
Clinical N (*N*, %)	
N0	384 (96)
N1	16 (4)
Clinical M (*N*, %)	
M0	344 (86)
M1	56 (14)
Followup (months, median, IQR)	36 (17–80)

IQR: interquartile range; *N*: number; TNM: tumor-node-metastasis staging.

**Table 2:** Relationship between clinical factors and overall survival in clear cell renal cell carcinoma.

Variable	Number of patients	Three-year overall survival rate (%)	95% CI	*P* value
Performance status				
0	368	89.9	198.6–248.9	0.003
≥1	32	72.9	77.5–151.1	
Gender				
Male	286	88.4	157.3–196.9	0.596
Female	114	85.8	217.2–267.1	
Hemoglobin				
≥LLN	329	92.1	191.8–250.9	0.004
<LLN	71	72.9	152–215	
Albumin				
≥LLN	282	90.3	188–254.1	0.010
<LLN	118	81.5	153.2–206.8	
BChE				
≥100 U/L	356	89.3	194.6–251.6	0.004
<100 U/L	44	77.7	101.6–154.8	
NLR				
≤2.5	287	90.2	187.5–244.0	0.457
>2.5	113	81.6	114.3–174.1	
Corrected calcium				
≤10 mg/dL	383	89.0	195.8–245.5	0.007
>10 mg/dL	17	64.5	65.5–155.8	
LDH				
≤1.5 × ULN	396	88.0	196.3–241.9	0.551
>1.5 × ULN	4	75.0	42.5–86.7	
C-reactive protein				
≤0.3 mg/dL	281	93.0	219.4–265.6	<0.001
>0.3 mg/dL	119	76.4	116.8–175.6	
T stage				
T1	261	93.0	233.8–274.3	<0.001
T2	41	88.6	101.1–22.6	
T3	88	74.4	126.1–181.3	
T4	10	77.1	36.9–71.6	
N stage				
N0	386	89.4	198.4–244.9	0.001
N1	14	45.3	36.7–100.7	
M stage				
M0	344	93.4	187.3–232.1	<0.001
M1	56	57.9	82.1–167.5	

CI: confidence interval; BChE: butyrylcholinesterase; LDH: lactate dehydrogenase; NLR: neutrophil-lymphocyte ratio; LLN: lower limit of laboratory's normal range; ULN: upper limit of laboratory's normal range; TNM: tumor-node-metastasis staging.

**Table 3:** Multivariate analysis in 400 patients with clear cell renal cell carcinoma.

Variable	Wald	95% CI	*P* value
M stage	27.784	0.085–0.324	<0.001
BChE	9.022	0.173–0.691	0.003
Age	2.444	0.963–1.004	0.118
Hemoglobin	1.979	0.287–1.228	0.160
N stage	1.226	0.268–0.562	0.268
T stage	0.846	0.413–4.179	0.846
C-reactive protein	0.352	0.402–1.629	0.553
Performance status	0.095	0.353–2.135	0.759
Corrected calcium	0.004	0.340–2.752	0.950
Albumin	0.003	0.501–2.081	0.954

CI: confidence interval; BChE: butyrylcholinesterase; TNM: tumor-node-metastasis staging.
